# Processes of increasing medical residents’ intrinsic motivation: a qualitative study

**DOI:** 10.5116/ijme.6250.1017

**Published:** 2022-04-29

**Authors:** Kazuki Tokumasu, Mikako Obika, Haruo Obara, Makoto Kikukawa, Yoshito Nishimura, Fumio Otsuka

**Affiliations:** 1 Department of General Medicine, Okayama University Graduate School of Medicine, Dentistry and Pharmaceutical Sciences, Okayama, Japan; 2 Department of General Internal Medicine, Okinawa Chubu Hospital, Uruma, Japan; 3 Department of Medical Education, Kyusyu University, Fukuoka, Japan; 4 Department of Medicine, John A. Burns School of Medicine, University of Hawai’i, Honolulu, USA

**Keywords:** Autonomy, intrinsic motivation, qualitative study, responsibility, self-determination theory

## Abstract

**Objectives:**

This study aimed to determine qualitatively how medical residents develop intrinsic motivation to learn and work in clinical training settings.

**Methods:**

This study was a descriptive qualitative study, which is widely used in healthcare research. We conducted a semi-structured interview aimed to explore key participants’ in-depth experiences and perspectives regarding intrinsic motivation. The authors interviewed seven postgraduate Japanese medical residents. The transcripts were analyzed using the sequential and thematic qualitative data analysis technique steps for coding and theorization, which entails coding steps from open to selective, writing a storyline using the final selective codes, and offering theories.

**Results:**

External stimulations (a self-handle environment and a near-peer role model) triggered the medical residents’ cognitive process (gap recognition, awareness, and internalization) to intrinsic motivation. The residents’ awareness of autonomy, responsibility, and independence played a vital role in this process. Furthermore, a psychological feeling of competence also reinforced their intrinsic motivation. Positive feedback and approval from attending physicians and patients’ gratitude promoted residents’ sense of competence.

**Conclusions:**

We illustrated a process for increasing medical residents’ intrinsic motivation. The intrinsic motivation was triggered by external stimulations (a self-handle environment and a near-peer role model), which caused the cognitive process: gap recognition, awareness of important attitudes as a doctor (autonomy, responsibility, and independence), and internalization. Since the first step of this process was an external factor, there are potential benefits of designing an appropriate training environment for increasing medical residents’ intrinsic motivation.

## Introduction

The importance of motivation is well known since it has been extensively researched in medical education. Motivation is a predictor for learning, academic success, persistence, a continuation of academic studies, and well-being.^1^^-^^3^ Among medical students, higher intrinsic motivation has been found to correlate with higher levels of meaning orientation, reﬂection in learning, academic achievement, cross-year peer tutoring experience, and intent to continue pursuing academic studies.^4^ Motivation can be classified as intrinsic or extrinsic. Intrinsic motivation refers to engaging in an activity for the pleasure or satisfaction that is inherent in doing the activity^5^^,^^6^ by virtue of an individual’s interests or the enjoyment he/she derives from the activity itself^7^ rather than for some separable consequence.^6^ Extrinsic motivation, on the other hand, is a construct that applies whenever an individual pursues an action to attain some separable outcome. Extrinsic motivation thus contrasts with intrinsic motivation in that the latter refers to doing an activity simply for enjoyment, while in the former, the individual seeks to derive some instrumental value.^5^

Research has established that motivation exists on a continuum, with intrinsic motivation at one end of the spectrum and amotivation (the absence of motivation) at the other, per self-determination theory.^5^ Extrinsic motivation entails different levels of self-determination; hence, it is composed of four stages of regulation: external, introjected, identified, and integrated.^5^^,^^6^^,^^8^ According to self-determination theory, intrinsic motivation is nurtured by the inherent psychological need for “autonomy,” “competence,” and “relatedness”.^6^ The need for autonomy is related to the feeling of acting on one’s own volition. The need for competence is related to feeling capable of achieving what one seeks to attain. Finally, the need for relatedness concerns feelings of belonging and connectedness in relation to others.^6^ Significant others often include parents, teachers, colleagues, and peers; in medical education and practice, this group can include patients.^7^

In the undergraduate setting, much research has been done on factors influencing motivation (i.e., support for autonomy, curriculum type, extent of responsibility, self-efficacy, selection procedure, assessment, rewards, knowledge acquisition, perceived task value, early patient contact, and well-being).^7^

In the postgraduate setting, doctors are motivated both to learn and to treat patients.^9^ Several factors that develop motivation were identified in previous research. Powerful experiences (i.e., challenges and major life events) and helpful relationships (characterized by openness, trust, and facilitative communication) were found to trigger introspection and thus provide motivation (in the form of the desire to understand and/or improve oneself) among a medical faculty.^10^ It was found that professional satisfaction, rewards, leadership, supervision support, and feedback influenced health workers’ motivation in Africa.^9^ It was also found that compared with training in a university setting, on-call duty in primary care settings created a greater sense of responsibility;^11^ interns seemed to be more motivated by their general practice training, perhaps due to their more robust sense of personal responsibility for patient care. A sense of personal responsibility is more essential to an intrinsic motivator than to an external one.^11^

However, there are few well-known means for increasing the intrinsic motivation of medical residents at the beginning of their growth phase as doctors in a clinical setting.  This study aimed to determine qualitatively how medical residents develop intrinsic motivation to learn and work in the clinical training setting.

## Methods

Study design and participants

We carried out a descriptive qualitative study^12^ and analyzed data using the steps for coding and theorization (SCAT)^13^ method with a social constructivism paradigm. Participants were selected by convenience sampling, which relies on the population that is available to participate. We analyzed each participant's qualitative data sequentially after conducting the interview and discussed the themes with co-researchers. We continued sampling and analysis until saturation was reached with no themes and concepts being generated from the data.^14^^,^^15^ These sampling and analyzing processes were continued from June 2016 to August 2019.

Seven medical residents participated: three from Hospital A and four from Hospital B. There were five male participants and two female participants, and all of them were Japanese. Their self-identified characteristics are shown in Table 1.The Ethics Committee of Okinawa Chubu Hospital (Approval number H27Chu Rin Sho Dai 46 Gou) and the Ethics Committee of Okayama University (Approval number Ken1603-044) reviewed and approved this study. As ethical considerations, a private room was prepared within the researchers’ facility to maintain participants’ privacy and psychological safety. All of the participants were given an explanation about the research objectives, their expected role, and the voluntary nature of participation. They were also informed that their decision to participate or decline participation would not affect any benefits or services they received. Each participant signed a written informed consent form and permitted audio-recording of the interview. The participants were also given assurance about confidentiality and anonymity of recordings, transcripts, and any behaviors observed during interviews, assurance about the voluntariness of participation and withdrawal, and that assurance information obtained would be used solely for the study.

**Table 1:** Self-identified characteristics

Participant	Self-identified characteristics	Interview time
#1	PGY-2, male, 34 years old, aspiring emergency room doctor, Hospital A	51 min+39 min (follow**-**up interview)
#2	PGY-2, male, 27 years old, aspiring general practitioner, Hospital B	68 min
#3	PGY-2, male, 26 years old, aspiring cardiologist, Hospital B	51 min
#4	PGY-4, male, 37 years old, belonging to internal medicine residency program, aspiring nephrologist, Hospital A	65 min
#5	PGY-3, male, 26 years old, belonging to internal medicine residency program, aspiring general internal medicine doctor, Hospital A	58 min+10 min (follow**-**up interview)
#6	PGY-2, female, 38 years old, aspiring surgeon, Hospital B	79 min
#7	PGY-2, female, 25 years old, aspiring internal medicine doctor, Hospital B	64 min

### Interviews

We conducted semi-structured interviews. At the beginning of the interviews, the participants were asked to provide demographic information (i.e., age, medical school and training, sex, etc.). After that, the participants were asked open-ended questions according to the interview guide (Appendix).

Based on the responses to the questions, the interviewer (KT) added specific questions to deepen interpretation of the origin of motivation and to detail the processes of increasing intrinsic motivation. Having worked as a physician at a university hospital, KT interviewed all of the participants in Japanese because KT was a well-trained interviewer for a qualitative study and had a good understanding of the research questions. KT did not handle, manage or assess participants’ training.

### Data collection methods

We used semi-structured interviews as a data collection method to access the participants’ personal perspectives and relevant experiences in order to facilitate a rich, detailed exploration of the research questions.^16^ All of the participants received an explanation of the semi-structured in-depth interview process and gave their consent to participate. The semi-structured in-depth interviews were conducted using the guiding questions shown in Appendix. Data were obtained via either the participant’s written responses or semi-structured interviews, lasting approximately 50 to 90 minutes. All interviews were recorded and transcribed. Follow-up interviews were conducted to explore the concept and interpret the experiences in detail.

### Setting

This study was conducted in Japan at two teaching hospitals (Hospital A and Hospital B). Japan is an appropriate place to conduct this research because of its advanced health care infrastructure and internationally recognized quality of medical education.^17^^,^^18^ Japanese residency programs have a proprietary clinical training system that requires at least two years to complete. During the first two years after medical school, medical residents in Japan rotate in several departments, including internal medicine, emergency medicine, surgery, pediatrics, obstetrics and gynecology, psychiatry, and rural medicine.^19^ After two years, some resident physicians choose to continue specialty training for three years.

#### Hospital A

Hospital A is a public 550-bed tertiary care teaching hospital with a large and active emergency department in central Okinawa. Most rotations include overnight on-call shifts in addition to daily patient care activities such as work rounds and outpatient clinics. Because Okinawa Prefecture has jurisdiction over many remote island clinics and hospitals, most residents go to the islands for several weeks of training.

#### Hospital B

Hospital B is a public 855-bed tertiary care teaching university hospital in central Okayama. The rotation collaborates with a regional core hospital (400 beds). Hospital B is linked to 53 regional hospitals, which enable residents to learn about rural medicine.

### Data analysis

Qualitative semi-structured interviews were conducted with the seven participants. The interviews were recorded and transcribed, and the transcripts were analyzed using the sequential and thematic qualitative data analysis technique SCAT,^13^ which is an effective method for analyzing small sets of data and can be easily employed by novices. This measure involves listing the segmented data within a matrix and then performing four coding steps in order: 1)  identification of words deserving focus within the data, 2) identification of words external to the text that can be used to restate the focus words, 3) identification of concepts external to the text that explain the focus words, and 4) a coding process using the themes and structural ideas that have arisen. Finally, the authors wrote a storyline using the final selective codes and offered theories.^20^^,^^21^

We chose this approach for its explicit analysis process, its integration of qualitative data analysis with theoretical coding, and its efficiency and validity of theorization based on relatively small-scale data with a social constructivism paradigm.^22^^,^^23^ The principal researcher (KT) performed each step of the analysis in Japanese, and as an independent auditor, a co-researcher (MO) read all of the transcripts and reconsidered each step of SCAT independently. Furthermore, co-researchers (HO, MK, YN and FO) checked all of the codes and the concepts. They also did a consensus discussion multiple times. Through the discussion, a new code emerged, and a code was revised from different points of view. All of the authors acknowledged new codes and concepts from transcripts during these iterative processes at the end of this analysis. Furthermore, we showed the results to five participants in order to confirm any discrepancies in data interpretation. This member checking process results in improvement of the trustworthiness of the qualitative data.^24^ The analysis was intended to assess dependability and confirmability^25^ and minimize confirmation bias. Finally, we identified the conceptual structure of the medical residents’ self-reported intrinsic motivation.

### Theoretical framework

We used Deci and Ryan’s self-determination theory, specifically their illustration of the motivation continuum^5^^,^^6^^,^^8^ which moves from a non-self-determined state (amotivation) to a self-determined state (intrinsic motivation), with intervening types of regulation as follows:

       1.   External regulation: behaviors are undertaken to satisfy an external demand or a contingent reward.

       2.   Introjected regulation: introjection involves internalizing a regulation without fully accepting it as one’s own. It is a relatively controlled form of regulation in which behaviors are undertaken to avoid guilt or anxiety or attain ego enhancements, such as protecting or bolstering one’s pride.

       3.   Identified regulation: identification reflects the conscious valuing of a behavioral goal or regulation, such that the action is accepted or owned as personally important.

       4.   Integrated regulation: integration occurs when the identified regulations are fully assimilated to the self, meaning they have been evaluated and brought into congruence with the individual’s other values and needs.

       5.   In self-determination theory, intrinsic motivation is built on the psychological need for autonomy, competence, and relatedness, the fulfillment of which intrinsically motivates a person to behave in a particular way. Our study was designed to reveal how medical residents develop intrinsic motivation through several processes and compare their respective positions on the motivation continuum in the clinical setting.

## Results

The self-handle environment (i.e., on-call duty or an emergency center) triggered residents’ perceptions of their own work as physicians. An environment in which medical residents cannot rely on attending doctors drives autonomous decision-making because they are faced with real patients who urgently need help. This urgency, compounded with the sense of being alone, comprised a self-handle environment that led the residents in this study to perceive themselves as having responsibility as doctors. Subsequent experiences of successful medical practice increased the residents’ sense of competence, thus increasing their intrinsic motivation for clinical practice and learning. According to Resident 6, making decisions under sociological, psychological, and restrictive time pressures drives the willingness to assertively undertake actions such as ordering several medical examinations and other related tasks. The resident reported feeling intrinsically motivated when the patient’s condition improved.

“I felt motivated after I dealt with an unexpected crash of one of my patients. Well, I got a call from the hospital in the middle of the night and then I rushed there and I was pretty much, well, a bit panicked. I somehow gave orders to the nurses and consulted on-call specialists that I thought necessary. Then I discussed with the patient what was going on and did some procedures. After I had a clear idea about what was going on and could stabilize the patient’s condition, I felt my motivation increased.…though I was still a resident physician, I really could see I was [working as] a doctor at the moment...I really felt the responsibility [as a doctor at that time].” (Resident 6: PGY-2, 38 years old, female)

Resident 4 said:

“Now [as a PGY-4], I can make an argument about a differential diagnosis based on my knowledge and experience [with my attending physicians]…[It is what I could not do a year ago, and I can see some improvement in my clinical skills. It is why] I like this work, and I would work harder and harder [to improve my skills further]…I think the sense of being more competent than before has motivated me a lot.”

The interviewer told the experience in detail that drove his motivation.

“There is an environment where I cannot rely on anyone, for example, when I am on a night cover. I can ask my attending physicians anytime but basically, I need to be independent and decide what to do on my own. Also, an extreme example is that I am on on-call duty at the hospital in a remote island . . . If I am not in that kind of situation [where I have to make a decision by myself], I feel spoiled or dependent. If I have attending physicians always with me, I will just wait for their decisions. Even if their assessments and plans are different from mine, I would just accept them and make orders without critical thinking. When I have to decide by myself, I would be serious about doing whatever [related to patient care].” (Resident 4: PGY-4, 37 years old, male)

This resident described his roots of intrinsic motivation. That was triggered by the self-handle environment where he could not rely on anyone such as a remote island setting. His intrinsic motivation for learning and working was increased as a doctor through responsibility for patient care.

Having a near-peer role model exerted a positive influence in terms of residents’ need to feel competent. Spending time with a well-trained senior doctor inspired the resident doctors to attain the same high level of performance that they observed in their respective role models and improved their sense of competence. Resident 3 described the near-peer model as an external stimulation, and through the process of internalization, that achieved the need to feel competent, finally, his intrinsic motivation increased. Resident 3 said:

“My wife is one grade above me, and she is working at a busy community hospital as a neurosurgery fellow. Thus, I fully understand the situation [and how busy it would be] . . . even if she is still a PGY-3 physician; for example, if you have any non-medical cases, she needs to manage the patients by herself. Seeing how she works, I knew I had to start learning now…Of course, I guess attending physicians would be helping her, but seeing PGY-3s need to work harder than I thought. I realized I need to accumulate a certain amount of knowledge and experience during my residency to be independent [before I become a PGY-3 physician]…Knowing how my wife has been working independently was very motivational for me, as I could image how I would behave next year and later.” (Resident 3: PGY-2, 26 years old, male)

Having a near-peer role model and witnessing her professional medical behavior allowed Resident 3 to recognize the importance of managing patients more meticulously based on concrete instructions; thus, he had a clear idea of what he should do in the future:

“When I started seeing how my wife worked, I felt [that my clinical ability was] terrible, so I started learning a lot . . . I definitely think my wife motivated me to learn more. Sometimes I see how she deals with inpatient consults or concerns about inpatients from the nurses over the phone. The queries from nurses are so detailed, for instance, they are asking by how many millimeters they should advance devices . . . and she answers these things in detail. By observing the conversations, I realized that a detailed understanding is fundamental [when it comes to patient care]…I realized I have to manage [patients] by myself…I did not get it just by being with my attending physicians. I got it [the need to be independent and meticulous] by having my wife [as a role model of patient care], who is the closest to me.” (Resident 3: PGY-2, 26 years old, male)

Furthermore, Resident 4 described the importance of having a near-peer role model from among the peer residents in the same work setting:

“When I was a PGY-1 resident, I got a sense about how I should behave as a PGY-2 looking at my uppers…for example, my uppers had their plans before they presented cases with attending physicians…Working with PGY-2s, I formulated my ideal image as an upper [resident]…Now I feel like I have been working hard to be the ideal upper.” (Resident-4: PGY-4, 37 years old, male)

The near-peer role model enhanced the ideal image of a doctor for this resident. Resident 4 reflected his ability as a doctor and then recognized the gap.

The motivational cognitive process started with recognizing the gap, leading to awareness and then internalization.

The medical residents recognized the gap between the competent doctors they aspire to be and their resident selves in the situations at hand. Understanding what they are not yet capable of doing and that they have not yet made their vision of being a doctor a reality induced them to recognize what they need to do, as evidenced in the following statement from Resident 5:

“I cannot work proactively by myself if I have anyone helping me [anytime]. A sense of responsibility [as a doctor] does not really come into play when there is someone who always helps you…As a third-year resident, we [as medical residents] have to go to a remote island [a few months for training]. When you go to a remote island, you have to take care of things [happening in the hospital] yourself. So, [after we are back to mainland Japan from the remote island, medical residents get a sense that] we have to manage patients proactively.” (Resident 5: PGY-3, 26 years old, male)

During the situations they described, medical residents perceived responsibility and autonomy, which are core values of being a doctor. This process explained important awareness as physicians. These perceptions, in turn, gave rise to a sense of personal responsibility and autonomy. Thus, the residents’ willingness to be independent as doctors emerged, as Resident 5 noted:

“I think I get the most sense of responsibility when working in the emergency department or on an internal medicine night float. Especially when I am on a night float, I need to see really sick patients when you are the only one who can help. . . I feel panicked at first in the situation, but feeling panicked is also a sense of responsibility to a certain extent. I feel panicked because I am responsible enough to believe that I have to deal with the situation by myself.” (Resident 5: PGY-3, 26 years old, male)

Resident 6 also supports the above details about awareness. The resident realized the responsibility as a doctor when she had to make decisions under sociological, psychological, and restrictive time pressures.

“…stabilize the patient’s condition, I felt my motivation increased…though I was still a resident physician, I really could see I was [working as] a doctor at the moment...I really felt the responsibility [as a doctor at that time].” (Resident 6: PGY-2, 38 years old, female)

This awareness (cognitions of responsibility, autonomy and independence) as a physician is linked to the next process.  The medical residents internalized their awareness of autonomy, responsibility, and independence; that is, they recognized that these are essential to being a doctor.

“If I am not in that kind of situation [where I have to make decisions by myself], I feel spoiled or dependent. If I have attending physicians always with me, I will just wait for their decisions. Even if their assessments and plans are different from mine, I would just accept them and make orders without critical thinking. When I have to decide by myself, I would be more serious about doing whatever [related to patient care].” (Resident 4: PGY-4, 37 years old, male)

The psychological need for competence may reinforce internalization. Furthermore, the internalization reflected the willingness to change behavior as a doctor who serves as both a learner and a worker.

“At the beginning of PGY-3…, I had no experience managing a patient with diabetic ketoacidosis on my own. One day, when I was on call, I had to manage a patient with diabetic ketoacidosis overnight semi-independently, following a protocol, and fortunately the patient got better. The fact that I took the initiative gave me competence [as a doctor]. I gained confidence from the fact that the patient got better and that I was appraised of the management during a morning round the following day [by the attending physician]. …I think I was able to acquire the style of learning and managing [patients] on my own thanks to the guidance and environment. These [the style of learning and managing patients on my own] are still very useful.…The fact that I could feel that I could progress, or that I had made progress, was an internal motivator.” (Resident 4: PGY-4, 37 years old, male)

In our results, the medical residents emphasized that fulfilling the psychological need for competence played a key role in increasing their intrinsic motivation.

Resident 1 described feeling competent after autonomously administering a successful treatment; the sense of responsibility that preceded treating the patient and the patient’s gratitude thereafter reinforced the efficacy of the motivation building process as well as the resident’s sense of competence, ultimately serving to increase intrinsic motivation:

“The case was a patient who came in with a foreign body in her ear. When I removed it, she was very happy, and then she asked me for my name. She said, “Oh, Dr. Something, thank you.  I remember your name now”. She was very grateful for me, and the sense of being appreciated made me very happy and motivated…Several days after that happened, I had more fun with work than usual. It is not that I don’t usually have fun with work, but I was able to work being more motivated than usual.” (Resident 1: PGY-2, 34 years old, male)

Positive feedback from attending physicians reinforced residents’ sense of competence, making them feel sufficiently intrinsically motivated, as evidenced in the following statements:

“I think I am most motivated when I receive positive feedback. Especially when I get positive feedback from my attending physicians giving me direct supervision, and also when I receive words of gratitude from patients, it really motivates me. And also when I notice something with patients that everyone else is not aware of, and I can report [the findings] to my attending physicians…” (Resident 2: PGY-2, 27 years old, male)

“If I’m proactive, attending physicians listen to me and start to rely on me. When they start to rely on me, it makes me feel like I’m doing a good job. And that makes me motivated.” (Resident 3: PGY-2, 26 years old, male）

In addition to attending physicians’ approval––specifically receiving attending physician’s positive feedback on appropriate assessments served to strengthen residents’ self-perceived competence––patients’ gratitude also reinforced residents’ feelings of competence, as evidenced by the following comment from Resident 7:

“There were times when [a patient] would say, “If you say so, I trust what you say.” Well, the fact that patients express trust in me motivates me even more. Another example is that if I explain findings to my patients with responsibility, they may feel my sense of responsibility at work and say “Doctor, I have no doubts about your decisions on my care.” The more motivated you are, the more you want to help your patients, and the more responsibility you have to help them, the more you study. After studying a lot, you can explain [patients’ conditions] to them with more confidence. And then, again, my patients may feel my sense of responsibility at work…” (Resident 7: PGY-2, 25 years old, female)

## Discussion

The findings presented in this paper are derived from a qualitative evaluation of medical residents. The generalizability of the study’s results to other settings should be treated with some caution. However, considering how the working and learning environments relate to one another and how external stimulations can motivate learning and working should provide a valuable chance to consider and reflect for those who plan and implement clinical education.

The results of this study showed some ways of intrinsic motivational processes among medical residents. The results highlight the source of (aspirational) physicians’ intrinsic motivation. It was triggered by immersion in a self-handle (autonomous decision-making) environment and access to a near-peer role model. Furthermore, external stimulations (a self-handle environment and a near-peer role model) resulted in process gap recognition, awareness of important attitudes as a doctor (autonomy, responsibility, and independence), and internalization. This motivational process is linked to intrinsic motivation for learning and working.

This process represented the cognitive process of increasing intrinsic motivation as medical residents. Based on motivational research, there are processes for increasing intrinsic motivation on the continuum from amotivation to intrinsic motivation, through extrinsic motivation in self-determination theory described in organismic integration theory.^8^ Amotivation is the state of lacking the intention to act, and extrinsic motivation is regulated (classified) in four steps (external regulation, introjected regulation, identified regulation, and integrated regulation). The medical residents' motivational cognitive process may be similar to identified regulation. Identified regulation involves a conscious valuing of a behavioral goal or regulation and accepting the behavior as personally necessary. Identification represents an essential aspect of the process of transforming external regulation into true self-regulation. When a person identifies with an action or the value it expresses, they, at least at a conscious level, are personally endorsing it. Thus, identifications are accompanied by a high degree of perceived autonomy. That is, identifications tend to have a relatively internal perceived locus of causality.^8^

Through the external stimulation provided by working in a self-handle environment, the medical residents recognized the gap between the doctors they aspire to be and their current capabilities, which led them to perceive strong cognitions of autonomy, responsibility, and independence as physicians. Therefore, medical residents need to be autonomous decision-makers in their clinical training environments as well as in the abovementioned cognitive processes, which refer to an automatic rather than a reflective system.^26^ For medical residents, it is possible that external stimulations (the self-handle environment and the near-peer role model) automatically drive the first step of perception with regard to important cognitions (autonomy, responsibility, and independence) as a physician, which are described in terms of awareness.

In our study, medical residents’ awareness was the crucial cognitive moment. The awareness included autonomy, which is key to building intrinsic motivation, as stated in self-determination theory literature.^5^^, ^^6^ Responsibility is also key to increasing intrinsic motivation. Moreover, self-satisfaction and self-efficacy have been found to be positively related during on-call duty in the primary care setting.^11^ When residents made a decision that resulted in success, attending doctors’ approval and patients’gratitude reinforced their internalized sense of competence, thus providing support for autonomy and facilitating the integration of behaviors.^6^ Competence contributes to the facilitation of internalization and the subsequent self-regulation of motivated activities in organismic integration theory.^5^^,^^6^ Medical residents perceived competence, which promoted internalization of an attitude as a physician and ultimately increased the medical residents’ intrinsic motivation. The psychological need to feel competent was reinforced by successful clinical experience. Additionally, autonomy and responsibility, which are crucial qualities for clinicians in postgraduate clinical settings, are powerful factors that bolster successful experiences. As young doctors, residents may perceive a considerable gap between their goals to contribute to patients’ management and their actual abilities.

Furthermore, the medical residents’ narratives revealed the importance of having a near-peer role model. The closer the role model is, the stronger is the cognitive process. Near-peer teaching appears to be beneficial for teachers and learners as well as for organizations.^27^ The proximity creates cognitive congruence (a small cognitive distance)^27^^,^^28^ meaning that residents are likely to consider themselves to have closer relationships with their peers than with attending physicians and faculty members, resulting in a powerful peer-assisted learning experience with the former.^29^ Role modeling delivered empowerment in terms of the psychological need for relatedness, per self-determination theory.

This article makes a novel point: the identification of perceptions of the cognitive processes of autonomy, responsibility, and independence can, from a physician’s perspective, be described as “awareness”. Awareness is triggered by an external environment where medical residents had autonomous decision-making and near-peer role modeling. Therefore, clinical education can support medical residents’ intrinsic motivation by regulating the external environment.

### Implications and future research

Medical residents’ motivational cognitive process described here may be suitable for several clinical educational environments. We emphasized the importance of external stimulations (a self-handle environment and a near-peer role model), which triggered the process. Clinical education can support medical residents’ intrinsic motivation and support residents’ clinical training positively by regulating the external environment (i.e., clinical educators can support setting an autonomous decision-making environment for residents).

For future research, by elucidating when and how one perceives their ideal attitude as a doctor, it will be possible to elaborate the cognitive process of increasing intrinsic motivation, which could be linked to professionalism.^30^ By describing their ideal images of themselves as doctors and the changes in the cognitive process between the ideal images and their own experience, the process of internalization and intrinsic motivation may be described further. Also, the motivational process of attending physicians could be a future area of research.

### Limitations

Our findings must be considered within certain methodological and cultural limitations. First, we revealed some possible processes for increasing medical residents’ intrinsic motivation. The findings were based on a small sample size of seven participants. However, the interviews lasted 50-90 minutes each, which was a reasonable length of time to interpret their motivational processes in depth from a social constructivist paradigm. Second, due to the difficulties involved in recruiting residents to participate, we used convenience sampling, which could have generated a self-selection bias. Furthermore, we selected the participants from a single nation (Japan) in East Asia with a traditional culture. Third, we could not clear the confirmation bias completely since one author conducted the interviews and six authors analyzed the qualitative data. To minimize this bias, we conducted an iterative process during analysis of the data. The principal researcher and the co-authors fully checked the transcripts and each step of analysis from different points of view, revisited the storyline and had many discussions. Finally, this study makes no clear distinction between motivation to learn and motivation to work because there were difficulties encountered in identifying and classifying the motivational differences between the two, since the medical residents were working in the clinical setting and learning at the same time.

## Conclusions

This study showed some ways of intrinsic motivational processes among medical residents. The results indicate the importance of the cognitive process and training environment of residents.

### Acknowledgments

We would like to thank all of the participants and the medical staff of Okinawa Chubu Hospital and Okayama University Hospital for their contribution to the management of the educational and training environment.

### Conflict of Interest

The authors declare that they have no conflict of interest.

## Supplementary material

Supplementary file 1 Appendix. Interview guide
