# Supplementary material for: Processes of increasing medical residents’ intrinsic motivation: a qualitative study
Source: Int J Med Educ. 2022 Apr 29;13:115–23. doi: 10.5116/ijme.6250.1017 (PMC9902172; doi:10.5116/ijme.6250.1017)
Supplement: Supplementary file 1 — Appendix. Interview guide [file ijme-13-115-S1.pdf]

Appendix

Interview guide

1. Can you recall which experiences motivated you as a doctor (medical resident)?

2. Please describe these motivating experiences in detail.

3. Could you tell me more about your interaction with the patients during the motivating experiences?

4. What were the specific points that motivated you during the experiences?
